# A Rigid-Flexible Coupled Lower Limb Exoskeleton for Enhancing Load-Bearing Ambulation

**DOI:** 10.3390/biomimetics10110757

**Published:** 2025-11-10

**Authors:** Yong-Tang Tian, Chun-Jie Chen, Xiao-Jun Wu, Wu-Jing Cao

**Affiliations:** 1 School of Mechanical and Electrical Engineering, Xi’an University of Architecture and Technology, Xi’an 710055, China; 2 Shenzhen Institutes of Advanced Technology, Chinese Academy of Sciences, Shenzhen 518055, China

**Keywords:** lower limb exoskeleton, rigid-flexible coupling, load transfer, metabolic cost

## Abstract

Lower limb exoskeletons significantly enhance human functionality. However, simultaneously improving the load capacity of these devices while reducing metabolic costs presents a major challenge in the industry. This paper presents a lower limb exoskeleton that integrates both rigid and flexible structures to facilitate active assistance and passive load transfer at the hip joint. The load transfer experiments were conducted with weights of 10 kg and 15 kg. During static standing, the load transfer rates were recorded at 90.48% and 69.70%, respectively. In dynamic walking, these rates decreased to 62.07% and 43.69%. Furthermore, in metabolic experiments involving a load of 15 kg, metabolic costs in the exoskeleton assistance modes OFF (Assist OFF) and exoskeleton assistance ON (Assist ON) were reduced by 8.3% and 21.61%, respectively, compared to the exoskeleton-free condition (NE). Furthermore, the Assist ON mode further decreased metabolic costs by 13.22% compared to the Assist OFF mode. These findings demonstrate that the rigid-soft coupled lower limb exoskeleton exhibits exceptional load transfer capabilities and effective assistance, highlighting its potential to enhance human performance in weight-bearing activities.

## 1. Introduction

Rigid lower limb exoskeletons (RLLEs) are extensively utilized due to their outstanding load-bearing capacity and high rigidity, which significantly enhance muscular strength and reduce metabolic costs [1,2]. They can be categorized into active and passive types based on the presence of an external power source [3,4,5,6]. Passive load-bearing exoskeletons, such as the non-anthropomorphic passive lower limb exoskeleton (NAPLE) developed by Zhan’s team, rely solely on mechanical structures to augment human load-bearing capacity. Weighing 6 kg, NAPLE utilizes a reconfigurable universal cylindrical rotation mechanism to adapt to various gaits, effectively addressing motion coordination issues inherent in anthropomorphic designs. Under a 30 kg load, NAPLE transfers 87.8% of the load to the ground while stationary and an average of 42.41% during ambulation [7]. However, while this class of exoskeletons facilitates load transfer through linkage mechanisms, they are incapable of providing active actuation. Moreover, the associated metabolic penalty incurred during operation has emerged as a significant constraint.

To mitigate the negative work imposed by exoskeletons on the human body and to provide proactive assistance, researchers have developed active load-bearing lower limb exoskeletons [8,9,10]. The Li team designed a RLLE featuring a constant-force suspension mechanism and adaptive compliant joints. Results indicate that this exoskeleton can reduce impact forces during movement. Compared to unassisted conditions, wearing the exoskeleton leads to a 10.95% reduction in metabolic rate while walking at 5 km/h and a 1.71% reduction during running at 9 km/h [11]. In rigid exoskeletons, appropriate assistive torque applied through motors aligned with the rotational centers of biological joints can similarly mitigate adverse effects in human-robot interaction [12]. This principle has motivated the widespread integration of series elastic actuators (SEAs) into exoskeleton joints [13,14,15]. However, SEA implementation may exacerbate joint misalignment risks and induce tangential forces/internal stresses within limbs that are particularly challenging to quantify and compensate for [16]. These parasitic forces can disrupt natural gait patterns, ultimately leading to user discomfort and elevated metabolic expenditure [17].

Furthermore, RLLEs frequently exhibit compromised wearability and user discomfort due to their inherently cumbersome structural design [18]. flexible exoskeletons exhibit lightweight architectures and enhanced compliance, which contribute to improved human-robot interaction while delivering active assistance. However, their inherent structural properties fundamentally limit effective load transfer capabilities [19,20]. However, they are unable to facilitate load transfer effectively [21]. Consequently, the integration of the advantages inherent in both rigid and flexible structures to develop exoskeletons with improved user ergonomics and superior load transfer capabilities has emerged as a prominent trend in the field. The research conducted by the Molinaro team reveals that exoskeletons composed of integrated rigid components and soft outer garments achieved a maximum reduction of 19.70% in metabolic cost during tests with an 11 kg load [22]. Despite achieving favorable metabolic costs, the exoskeleton does not make direct contact with the ground; instead, it transfers load through the calf. This design increases the inertia of the exoskeleton’s leg rods and heightens the requirements for binding. The transferred load is concentrated on the calf, thereby augmenting its burden and diminishing the exoskeleton’s capacity for effective human-machine interaction. To address the challenge of effective load transfer in exoskeletons, the co-author Cao’s team and Yu’s team independently incorporated footrest designs into their respective systems, enabling the transfer of gravitational loads to the ground. Empirical evidence from Cao’s team demonstrates that their hybrid rigid-soft exoskeleton achieves load transfer rates of 88% during static standing and 67% during ambulation while simultaneously providing active assistance. However, this study reported a relatively modest metabolic cost reduction of only 12.8%, and its experimental validation lacked comprehensive assessment of the hip-knee joints and a modular exoskeleton design [23]. Concurrently, Yu’s team reported a more substantial 13.1% reduction in metabolic cost; nevertheless, their work did not include an evaluation of the device’s load transfer performance [24].

To tackle the issues of reducing metabolic consumption and optimizing load transfer, our team has previously developed a series of exoskeleton prototypes to address these challenges [25,26]. In our prior work [25], we detailed a transmission system utilizing a Bowden cable to efficiently transfer assistive forces from a remote actuator to the thigh; assistance torque at the hip joint is generated as the motor retracts the cable, effectively shortening the distance between the winch and the thigh anchor point. Furthermore, to address issues of inertia and stability during load-bearing locomotion, we proposed in [26] the use of a fully enveloping woven fabric interface to secure the exoskeleton, which was empirically validated to effectively minimize relative sliding between the structural leg rods and the human body. This paper reports on the continued development and comprehensive evaluation of a further optimized exoskeleton system based on these prior prototypes. Empirical evidence indicates that the present system exhibits pronounced advantages in synthesizing high load-transfer capacity, substantial metabolic cost reduction, and enhanced human-machine interaction. Notably, it demonstrates the unique capability to reduce the user’s metabolic cost even in a passive state—a distinctive characteristic rarely observed in other exoskeleton systems. The primary contributions of this paper are as follows:

Leveraging the inherent advantages of integrating rigid and flexible structures, we developed the rigid-flexible coupled lower limb exoskeleton (RSCLLE) and implemented a modular design for key components, including the hip, knee, and ankle. Furthermore, we elucidated its operational principles, providing a comprehensive understanding of its functionality. Comprehensive testing of the exoskeleton’s load transfer capabilities and metabolic expenditure performance has been conducted, providing evidence of its substantial efficacy in assisting users during loaded walking scenarios.

The remainder of this paper is organized as follows. Section 2 delineates the overall architectural design of the novel exoskeleton system. Section 3 elaborates on the experimental design and implementation procedures. A systematic presentation and analytical discussion of the experimental results are provided in Section 4. Section 5 critically examines the scholarly significance and practical implications of this study. Finally, Section 6 concludes the paper by summarizing the principal findings and outlining promising avenues for future research.

## 2. Design of Exoskeleton Structures

### 2.1. Mechanical Structure and Driving System

**Figure 1:**
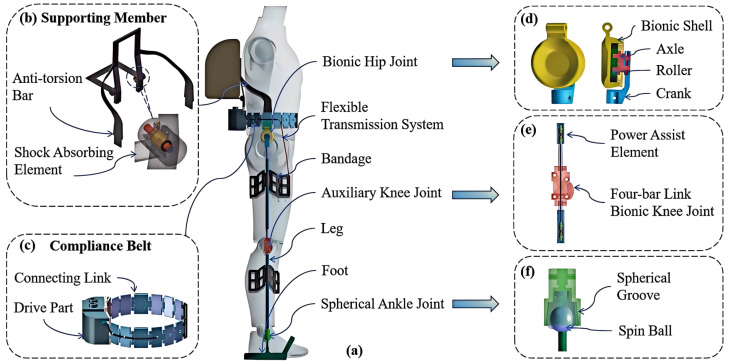
Main Components of the RFCLLE. (**a**) Component Layout. (**b**) Load-Bearing Support Structure. This structure comprises a load-bearing frame, an anti-torsion bar, and shock-absorbing elements, each integral to the overall stability and functionality. (**c**) Adaptive Belt. (**d**) Hip Joint. (**e**) Knee Joint. This component features a four-bar bionic structure in conjunction with elastic elements, optimizing mobility and providing essential support during knee extension. (**f**) Ankle Joint.

As shown in Figure 1a, the RFCLLE is primarily designed to assist and transfer weight during load-bearing walking. In contrast to conventional designs, the RFCLLE incorporates a novel integration of a back support element and an anti-torsion bar to provide primary load-bearing support. To enhance the stability of the system, a wearable vest is integrated into the waist belt. Shock-absorbing elements are incorporated at the load-bearing points to mitigate the impact on the human body during movement. Additionally, the anti-torsion bar works in conjunction with fulcrums on both sides of the waist belt to counteract torque and improve load transfer efficiency (Figure 1b). The waist belt is constructed from rigid links, allowing for adjustments based on the wearer’s body shape. The combination of links of varying sizes ensures proper alignment of the hip joint and facilitates load transfer to the ground via the connected rigid lower limb (Figure 1c). The modular lower limb system comprises a hip joint, an adjustable leg rod, a knee joint, a spherical ankle joint, a supportive foot structure, and flexible woven fabric binding interfaces. The bio-inspired structure of the hip joint and the energy-storing mechanism integrated into the knee joint collectively constitute the innovative design of the lower limbs.The hip joint connects to the chain link of the waist belt via a bio-inspired structure, ensuring anatomical conformity while minimizing interfacial gaps. Particularly noteworthy is the incorporation of an external crank, which significantly reduces the distance between the leg rod and the wearer’s body, thereby effectively mitigating the risk of hip joint misalignment in both the sagittal and coronal planes (Figure 1d). Figure 1e illustrates the innovative design of the knee joint. (1) A four-bar bionic structure that optimally simulates knee joint motion along a desired trajectory, while satisfying the conditions of flexion-extension angles and torque, thereby facilitating multi-rotational center movements of the exoskeleton in the sagittal plane [27]. The lengths of the components in the four-bar linkage are determined through the analysis of rotational positions using the Freudenstein equation [28]. This bionic structure has been validated in rehabilitation devices, effectively minimizing the relative motion between the exoskeleton and the human body [29]. (2) an assistive element, which includes two elastic components located in the thigh and calf rods, respectively, connected by a steel wire rope. When the knee joint flexes, the elastic components compress and store energy; during extension, they relax to provide assistance. When the user is standing, the elastic components remain relaxed. When the spherical ankle joint is in a standing position, the friction between the top of the groove and the rotating ball increases, achieving self-locking to ensure that the load is transferred to the ground. During the swing phase, the rotating ball can move horizontally within the groove and rotate to a certain angle in both the coronal and sagittal planes (Figure 1f). The RFCLLE discussed in this paper features adjustable calf and thigh rods, accommodating wearers ranging from 165 cm to 185 cm in height. All components of the exoskeleton are designed to be lightweight, with a total mass of approximately 5 kg. The advantages of the proposed RFCLLE over existing technologies are manifested in three key aspects: (1) The incorporation of an anti-rotation bar significantly enhances operational stability by minimizing relative sliding and misalignment with the human body, thereby effectively mitigating the adverse effects of external loads. (2) A modular architectural design enables rapid donning/doffing procedures and facilitates swift, precise dimensional adjustments for optimal user adaptation. (3) The bio-inspired knee joint integrates an energy-storing assistive mechanism that harnesses gravitational potential energy during flexion and releases stored energy to provide assistance during the extension phase.

**Figure 2:**
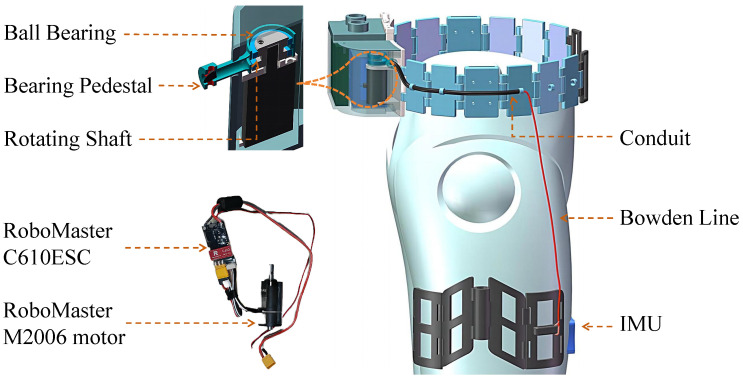
Drive System Structure.The layout of the Bowden cables is optimized using structures such as bearings and guide tubes to enhance cable efficiency. Compliant links increase the anthropomorphism of the exoskeleton, allowing it to accommodate a wide range of body types.

The flexion of the hip joint in the sagittal plane is primarily controlled by the iliopsoas and quadriceps femoris muscle groups [11,16]. To reduce muscle consumption in the hip joint during movement, this study integrates the driving design concept of the flexible wearable exosuit with the RFCLLE, as illustrated in Figure 2. The motor, development board, and power supply are housed within the rear belt and connected to an anchor point located on the front of the thigh, furthest from the hip joint axis, via a Bowden cable. When the hip joint flexes, the torque generated by the motor assists the muscle groups through the anchor point. Conversely, when the hip joint extends, the motor reverses direction, allowing the Bowden cable to return to its initial position under the influence of the leg’s gravity. If the motor reverses too quickly, the Bowden cable may not release in time, resulting in excessive load that could potentially cause the cable to break or damage the motor, thereby posing a risk to the user. To address this issue, we designed a specialized bearing support that incorporates two bearings positioned on either side of the shaft to minimize excess clearance. Additionally, this bearing support serves a guiding function, enhancing the smoothness of the cable retraction process.

The torque at the hip joint is influenced by various factors, including individual velocity and body weight. Consequently, the design of the assistive system is informed by the hip angle and torque profiles reported in the Winter dataset. At a walking speed of 4 km/h, the hip joint torque typically ranges between 30 and 50 N·m. When the target assistive torque is set to 50% of the human torque during ambulation, the maximum assistive torque provided by the actuator is 25 N·m [30]. The motor employed in this system (RoboMaster M2006, DJI, China) features a reduction ratio of 36:1 and is coupled with an electronic speed controller (ESC) (RoboMaster C610), capable of delivering a maximum continuous output torque of 1 N·m. With a winch radius of 0.08 m, this configuration permits a maximum output force of 125 N. To address efficiency considerations, the peak allowable assistive force is capped at 100 N. The output force application point of the RSCLLE is located 0.25 m from the hip joint, and the maximum output torque is 25 N·m, thereby adequately fulfilling the requirements for assistive walking under load conditions.

### 2.2. Control System

**Figure 3:**
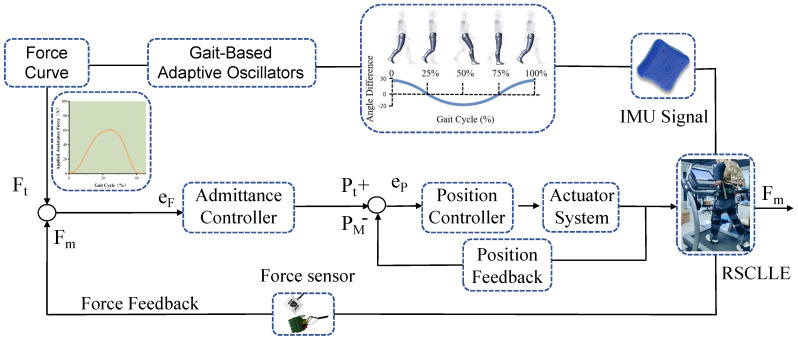
System Control Framework. The motion sensors consist of two inertial measurement units (IMUs) installed on the anterior side of the thigh, just above the knee joint. Two force sensors are mounted at the ends of the Bowden cables to provide force feedback. The sinusoidal torque curve is derived from previous work and serves as a reference for the output torque.

To provide appropriate assistive forces to the human body during motion, this study employs a gait-based adaptive oscillation (AO) method to estimate the percentage phase of walking. The gait-based adaptive oscillator (AO) system, featuring a dual-layer AO architecture, delivers accelerated convergence, enhanced estimation accuracy, and improved stability in gait phase prediction. During continuous gait phases, the proposed gait-based AO matches or surpasses the precision level of conventional AO methods while significantly reducing the synchronization time during non-steady-state walking. This approach does not require model training and enables the synchronization of exoskeleton assistance within two strides, thereby not only reducing the gait learning time but also enhancing the accuracy of predictions accordingly (For detailed research and performance parameters pertaining to gait-based Active Orthoses (AO), readers are referred to prior studies in which some of the authors have participated [31]). Based on the hip joint torque profile originally proposed in Reference [32], the present study introduces an optimized torque curve that not only effectively reduces metabolic consumption but also minimizes the risk of human injury due to excessive torque loading. Finally, the force feedback mechanism and admittance control are integrated to form the control strategy for the entire exoskeleton system, as illustrated in Figure 3.

When the RFCLLE is activated, the hip joint angle is initially assessed via the IMU. Subsequently, the assistance force $F_{t}$ is determined in accordance with the current gait phase and in conjunction with the torque curve.The force sensor integrated within the transmission system transmits the measured assistive force $F_{m}$ to the admittance controller, which generates the requisite desired motor position $P_{t}$ based on the error $e_{F}$ between $F_{t}$ and $F_{m}$. During operation, should the feedback position signal $P_{M}$ deviate significantly from the desired position $P_{t}$, the position controller promptly adjusts the motor to correct this discrepancy. The assistive force generated by this strategy is dynamically calibrated to accommodate variations in the user’s gait characteristics, thereby effectively minimizing metabolic costs.

Throughout the gait cycle, the fluctuation in the desired force $F_{t}$ is intricately linked to the time derivative of the hip joint angle. The mathematical expression for the assistive force profile is given by Equation (1) [25], where the phase parameter ϕ is defined as a continuous, periodic function that linearly increases with time and resets modulo 2π at the completion of each gait cycle. This formulation ensures periodic behavior aligned with the gait cycle while maintaining mathematical continuity.

(1) $$F_{t}=F_{m}\cdot \left(n+\frac{1}{2}\sin\left(\frac{\pi }{a}\phi -\frac{\pi }{b}\right)\right)\cdot c$$

In this equation, $F_{m}$ denotes the actual force output by the motor, while the gait phase ϕ is defined as ϕ = 2π*t*/*T*, where *t* represents the elapsed time and *T* corresponds to the duration of a full gait cycle. The parameters π/*a* and π/*b* function as scaling factors that modulate the frequency characteristics and phase shift of the force profile, whereas the parameter *n* defines the baseline force offset. The scaling factor *c* integrates biomechanical weighting coefficients derived from individual physiological attributes (e.g., body mass, muscle strength) and external load conditions, thereby ensuring system adaptability across diverse users and operational scenarios.

The position inner loop delivers essential positional feedback to the motor system, thereby significantly enhancing the precision of assistive force control. The admittance controller operates as the principal regulatory mechanism for the entire system, adeptly processing gait parameters to attain the desired motor positioning. Its operation is illustrated as shown in Equation (2):

(2) $$P_{t}=K_{p}\cdot e_{F}+k_{d}\cdot \frac{∆e_{F}}{∆t}$$

In Equation, $P_{t}$ signifies the target position of the motor, while $K_{p}$ and $K_{d}$ correspond to the proportional and derivative controllers, respectively. Furthermore, $e_{F}$ and $\frac{∆e_{F}}{∆t}$ denote the current force error and its differential error, respectively.

## 3. Experimental Design

To assess the comprehensive performance of the RFCLLE under load conditions, we recruited eight male participants to engage in experiments examining load transfer and metabolic costs. All experiments were conducted under identical environmental conditions to mitigate the influence of external variables. Recognizing that the subjects’ proficiency with the exoskeleton could influence the level of assistance provided, all participants underwent training on the proper techniques for wearing and utilizing the exoskeleton prior to the experiments [33,34]. Before the experiments, informed consent was obtained from all participants. The experimental protocol was approved by the Medical Ethics Committee of the Shenzhen Institutes of Advanced Technology (SIAT) under Application No. SIAT-IRB-240415-H0741.The final results were presented as mean ± standard error of the mean (SEM) and analyzed using a two-sided paired *t*-test with Holm-Šidák correction, with significance set at p≤ 0.05. The physiological parameters of the subjects are detailed in Table 1.

### 3.1. Experiment 1

**Figure 4:**
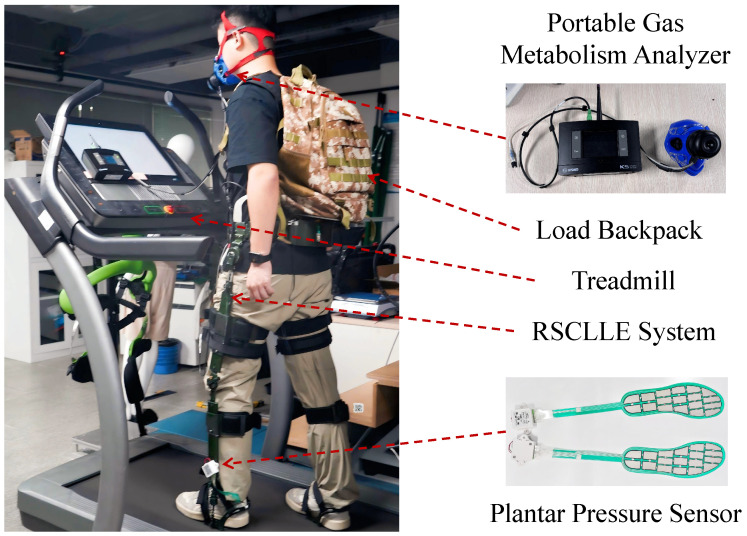
The subjects conducted two experiments: (1) a load transfer experiment using plantar pressure sensors, and (2) a metabolic cost test using a portable gas metabolism analyzer.

In this experiment, subjects carried varying loads for testing, which comprised two stages: quiet standing (QS) and dynamic walking (DW), As shown in Figure 4. Prior to the experiment, participants wore the RFCLLE, the RX-ES42-18 plantar pressure sensor (The sampling frequency was 10 Hz), and a weighted backpack to undergo a brief adaptive training session. During the experiment, once the readings from the plantar pressure sensors stabilized, participants were instructed to stand quietly for two minutes. Following this, the average value $X_{QS}$ of the collected data is utilized for the calculation of load transfer rates. Subsequently, the aforementioned procedure was adopted, directing participants to walk on the treadmill at a speed of 4 km/h for a duration of two minutes. The average value $X_{DW}$ obtained during this period was also integrated into the calculation of the transfer rate. Upon completing the experiment with this load, subjects were allowed a 15-min rest period before proceeding to the next set of experiments. The relationship for load transfer rate was presented in Equation (3):

(3) $$\eta =\frac{X-\alpha \cdot \bar{X}}{g_{2}}\cdot 100\%$$

In the formula, *X* represents the sum of body weight $g_{1}$, load $g_{2}$, and exoskeleton weight $g_{3}$, $\bar{X}$ represents the average values of data recorded by pressure sensors in the QS or DW state while the exoskeleton is being worn.

This study incorporates a bodyweight-dependent gain parameter α, to compensate for measurement inaccuracies in plantar pressure sensors. The calibration of α was performed under conditions where the subject was unloaded and not wearing the exoskeleton. The pressure-resistance characteristic of the plantar pressure sensor is defined by the following Equation (4), where *F* represents the sensor reading and *x* denotes the reciprocal of resistance. Let $g_{1}$ be the true body weight of the subject under zero external load, and ${\bar{g}}_{1}$ be the body weight measured by the pressure sensor under the same unloaded conditions. By substituting $g_{1}$ and ${\bar{g}}_{1}$ into the aforementioned pressure characteristic equation and solving the simultaneous equations, the analytical expression for the gain parameter α, namely Equation (5), is derived. Owing to the variation in body weight among different subjects, the calibrated gain parameter α also varies accordingly. To ensure the accuracy of the acquired data, the gain parameters for both the Quiet Standing (QS) and Dynamic Walking (DW) conditions were calibrated independently.

(4) F=k·x

(5) $$\alpha =g_{1}/{\bar{g}}_{1}$$

### 3.2. Experiment 2

Assessing the metabolic cost of exoskeleton-enhanced exercise has been established as a standard method for determining the energy expenditure of the wearer [33,35]. In Figure 4, the metabolic costs of exercise were evaluated under three conditions: no exoskeleton (NE), exoskeleton assistance turned off (Assist OFF), and exoskeleton assistance turned on (Assist ON), using a portable gas analysis system (K4b2, Cosmed, Roma, Italy). Initially, subjects stood still without any additional weight, and resting metabolic data were collected for 10 min during a steady phase. Subsequently, subjects walked on a treadmill at a speed of 4 km/h while carrying a load of 15 kg, with real-time metabolic data collected during the steady phase for 10 min. Following this portion of the experiment, subjects were allowed a 15-min rest period before proceeding to the next set of experiments. The metabolic rate was calculated using the modified Brockway formula [36], as presented in Equation (6):

(6) $$E=\left(16.89\cdot VO_{2}+4.84\cdot VCO_{2}\right)/\left(60\cdot W\right)$$

In the aforementioned equation, *E* is denoted as energy expenditure measured in watts per kilogram (W/kg). The variables $VO_{2}$ and $VCO_{2}$ denote oxygen consumption and carbon dioxide production, respectively, while *W* indicates the body weight of each individual.

## 4. Experimental Results

### 4.1. Load Transfer

**Figure 5:**
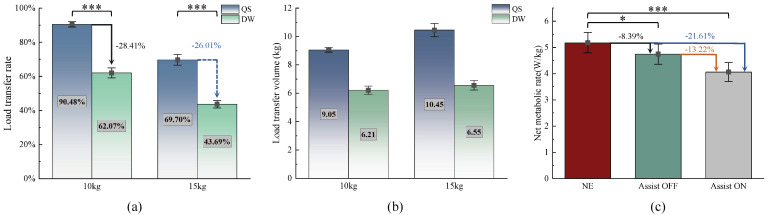
Experimental Results. (**a**) Load transfer rate. The load transfer rates under dynamic walking and static standing conditions were provided for loads of 10 kg and 15 kg. (**b**) Load transfer amounts. (**c**) Metabolic cost. The metabolic consumption of participants under a 15 kg load was analyzed across three conditions: NE, Assist OFF, and Assist ON.

Table 2 presents the load transfer rates of the exoskeleton under different experimental conditions. When the initial load weight is set at 10 kg, the average load transfer rates for the exoskeleton in the two states are 90.48 ± 1.59% (SEM) and 62.07 ± 2.90% (SEM), respectively. As illustrated in Figure 5a, the load transfer rate during quiet standing (QS) is 28.41% higher than that during dynamic walking (DW) (*p* = 0.00017). However, when the load weight increases to 15 kg, the load transfer rates decrease to 69.70 ± 3.15% (SEM) and 43.69 ± 2.18% (SEM), with the QS state still exhibiting a 26.01% higher transfer rate compared to DW (*p* = 0.00008). These results indicate that dynamic walking significantly impairs the load-bearing capacity of the exoskeleton’s legs, preventing it from transferring a greater load to the ground. Figure 5b illustrates the transfer amounts of the exoskeleton under 10 kg and 15 kg loads in both states, recorded as 9.05 ± 0.16 (SEM) kg and 6.21 ± 0.29 (SEM) kg, respectively, alongside 10.45 ± 0.47 (SEM) kg and 6.55 ± 0.33 (SEM) kg for each condition. Notably, while the load transfer rates decrease with increasing load weight in both states, the transfer amounts exhibit varying degrees of increase. This phenomenon may be attributed to exacerbated deformation of the soft binding interfaces in the exoskeleton under elevated load conditions, thereby amplifying relative sliding between the leg linkages and the human body. All comparisons of transfer rates demonstrate significant differences, underscoring the statistical relevance of the findings.

### 4.2. Metabolic Cost

The net metabolic cost of walking was calculated by subtracting the resting metabolic cost during quiet standing (QS) from the total metabolic cost during dynamic walking (DW). To minimize the influence of body weight on these calculations, the metabolic cost was standardized relative to each individual’s body weight. The details of the metabolic costs for the subjects are presented in Table 3. Notably, variations in metabolic costs were observed under the same conditions, which may be attributed to the physiological parameters and daily activity levels of the participants. When walking with a load of 15 kg, the average net metabolic costs for the three conditions—no exoskeleton (NE), exoskeleton assistance turned off (Assist OFF), and exoskeleton assistance turned on (Assist ON)—were 5.177 ± 0.387 W/kg, 4.742 ± 0.387 W/kg, and 4.060 ± 0.361 W/kg (Mean ± SEM), respectively.

The comparison of metabolic costs is illustrated in Figure 5c. The metabolic cost under the Assist OFF condition exhibited a decrease of 8.39 ± 2.74% (SEM; *p* = 0.03171) compared to the no exoskeleton (NE) condition. This finding suggests that the benefits conferred by load transfer through the RFCLLE surpass the metabolic costs associated with the exoskeleton itself. In this context, the RFCLLE demonstrates enhanced efficacy relative to other exoskeletons designed with load transfer capabilities. Moreover, the metabolic cost under the Assist ON condition was lowered by 21.61 ± 3.46% (SEM; *p* = 0.00039) in comparison to NE, a result attributable to the synergistic interaction between the mechanical structure and the motor system, which collaboratively provide assistance. Additionally, when comparing the Assist ON condition to Assist OFF, the net metabolic cost decreased by 13.22 ± 2.47% (SEM; *p* = 0.00069), indicating that the transmission design and assistance strategy we proposed can significantly mitigate metabolic costs. The observed reductions in net metabolic cost are statistically significant, further underscoring the considerable potential of the RFCLLE to enhance human performance in weight-bearing activities.

## 5. Discussion

This paper presents a rigid-flexible coupled lower limb exoskeleton (RFCLLE) that facilitates passive load transfer while providing active assistance for hip flexion and extension through a gait-based adaptive oscillator methodology. The exoskeleton demonstrates commendable efficacy in both load transfer and metabolic cost reduction. Load Transfer refers to the process of partially or completely redirecting loads—which are initially borne by specific anatomical regions (primarily the shoulders, back, and legs)—to the ground through an exoskeleton structure. This transfer is quantified by comparing the values measured by plantar pressure sensors with and without exoskeletal assistance. Metabolic Cost describes the rate at which the human body expends energy to perform a specific task (such as walking or load-bearing), typically expressed in Metabolic Equivalents or as power consumption per unit body mass (Watts/kilogram, W/kg). It is measured using systems such as the K5 metabolic analyzer under both assisted (exoskeleton-on) and unassisted conditions, with the percentage change calculated according to established formulae.

Load Transfer: As shown in Table 2, when the load is increased to 15 kg, the load transfer rates in both quiet standing (QS) and dynamic walking (DW) conditions exhibit a decline. This phenomenon may be attributed to the exacerbated deformation of the soft binding interfaces in the RFCLLE under elevated load conditions, which consequently amplifies the relative sliding between the exoskeleton and the human body. Furthermore, variations in participants’ body types may influence the extent of the decline in load transfer capacity, leading to differences in performance among subjects. Notably, Subject 3 achieved a load transfer rate at 15 kg that surpassed that of other participants, even exceeding their performance at 10 kg. This phenomenon may be attributed to mechanical locking occurring at the knee joint under elevated load conditions, which amplifies the interaction forces between the leg linkage and the human body, consequently inducing localized signal saturation in the plantar pressure sensors due to excessive stress concentration. Under a 10 kg load, this participant had a static standing transfer rate of 81.70%, significantly lower than that of others. This discrepancy may be associated with their slender physique, which could render them more susceptible to misalignment with the exoskeleton, ultimately resulting in decreased load transfer efficiency.

Metabolic Cost: The differences in metabolic cost are closely tied to individual cardiovascular fitness. As illustrated in Table 3, Subject 4 and Subject 5, being fitness enthusiasts, likely possess superior cardiovascular function, allowing them to achieve lower metabolic expenditures. Subject 7 exhibited a marked reduction in metabolic consumption under the Assist ON condition, which may be attributed to a loose oxygen mask causing respiratory leakage. Conversely, Subject 8 demonstrated a metabolic expenditure in the Assist OFF state that exceeded the normal exertion (NE) baseline. This observation is consistent with findings from previous studies.For instance, Reference [10] reported that compared to the NE and Assist OFF conditions, the Assist ON mode resulted in reductions of metabolic cost by 20.85% and 36.56%, respectively. Similarly, in [21], under the Assist ON condition, metabolic cost was reduced by 14.88% and 22.03% relative to the NE and Assist OFF conditions, respectively. This indicates that, in the absence of active assistance, the RLLE faces challenges in effectively reducing the user’s metabolic costs due to structural limitations and weight constraints. A reduction in metabolic cost can only occur when the benefits of load transfer outweigh the metabolic penalties associated with the weight of the RLLE itself. This finding underscores the importance of balancing load transfer efficiency with the inherent weight of the device in exoskeleton design to optimize metabolic performance.

Comparison with Existing Work: Table 4 provides a detailed comparative analysis of the proposed exoskeleton and existing lower limb exoskeletons in terms of assistive capability and metabolic cost. The RSCLLE demonstrates a significantly superior load transfer capability while standing, achieving 90.48%. This outstanding performance indicates that the RSCLLE effectively distributes the load while supporting the user, greatly enhancing stability and comfort. During gait walking, the load transfer efficiency of the present exoskeleton was measured at 62.07%. Although this value is marginally lower than the 67.00% reported by co-author Cao [23] under identical conditions, it represents a substantially superior performance when compared to the exoskeletons proposed by Zhou and Zhan—which provide only passive support, with maximum transfer efficiencies of 24.60% and 42.41%, respectively [5,7]. Furthermore, the RSCLLE has successfully integrated active assistance with load transfer capabilities, thereby delivering more comprehensive support to the user.

The RFCLLE also exhibits significant effectiveness in reducing metabolic costs. Under the Assist OFF condition, the RFCLLE achieved an 8.39% reduction in metabolic cost relative to the normal effort (NE) baseline. This observed reduction is substantially greater than the 3.7% decrease reported in a prior study by co-author Cao [23]. In contrast, other exoskeletons of the same category failed to achieve any reduction in metabolic cost under identical conditions. The finding underscores that the RFCLLE possesses a superior capability for attenuating metabolic cost, even in its passive assistance mode, highlighting its potential value for daily applications. Under the Assist ON condition, the RFCLLE achieved a significant 21.61% reduction in metabolic cost through the synergistic interaction between its mechanical structure and motor system, demonstrating performance superior to most comparable exoskeletons. Although the exoskeleton reported by Lee et al. in Reference [10] yielded a marginally higher reduction (22.03%) under identical conditions, its non-anthropomorphic structural design induced substantial impact during gait, adversely affecting user experience. In contrast, the RFCLLE maintained high energy efficiency while simultaneously ensuring natural movement and comfort, indicating better comprehensive performance. Under the same experimental conditions, the exoskeleton previously proposed by co-author Cao et al. attained only a 12.8% metabolic reduction [23]. The considerable improvement shown by the RFCLLE underscores the advantages of its innovative design, including mechanical structure, power transmission, and assistance strategy. Furthermore, supported by the motor system and assistance strategy of the RFCLLE, users can achieve a minimum 13.22% decrease in metabolic expenditure. This notable advancement provides critical insights for future exoskeleton design and further validates the potential of active assistance in reducing human energy consumption.

## 6. Conclusions

This study demonstrates that the Rigid-Flexible Coupled Lower-Limb Exoskeleton (RFCLLE) not only facilitates passive load transfer but also provides active assistance for hip flexion through its drive system. Results from two experimental groups confirm that the exoskeleton exhibits favorable performance in both load transfer and metabolic cost reduction, validating its advantages during weight-bearing ambulation. However, several limitations of this work must be acknowledged. First, the participant cohort consisted exclusively of individuals from a single, healthy population, which restricts the generalizability of the findings. Given the documented differences in musculoskeletal structure and metabolic response between males and females, as well as the variations in motor control and muscle function between younger and older adults, the optimal assistance parameters and efficacy outcomes identified in this study may not be directly generalizable to female or elderly individuals. To systematically address this limitation and enhance the external validity of the RFCLLE, future work will involve recruiting participants diverse in sex, age (including middle-aged and older adults), and body mass index (BMI) to better represent the potential user demographics for exoskeleton technology. We will investigate whether key parameters, such as assistance magnitude and timing, require adjustment across different demographic groups and explore personalized adaptation strategies for specific populations, such as women and the elderly. Second, the experiments were conducted solely on a treadmill, failing to replicate the complexities of walking on natural terrain. While this controlled experimental setup facilitates the reduction of variables and enables a preliminary validation of the exoskeleton’s core performance, it should be noted that such conditions inevitably limit the generalizability of the research findings. Specifically, the inherent biomechanical differences between treadmill walking and overground ambulation may lead to a scenario where the optimal assistance parameters identified in this study could prove suboptimal or require significant recalibration when deployed in real-world environments. In future investigations, we will evaluate the performance of the RFCLLE in real-world settings across varied terrains, including slopes, stairs, and uneven ground, to study whether the assistance parameters require recalibration for different walking conditions. Third, the load measurement relied on plantar pressure sensors, whose variation characteristics may influence data accuracy. Future efforts will focus on implementing more precise calibration of compensation parameters based on the sensors’ pressure profiles to improve the accuracy of the assistance parameters. Despite these limitations, by integrating previous research with our experimental data, we further confirm that the practical value of a wearable lower-limb exoskeleton is realized only when the assistance benefit it provides to the user exceeds the metabolic cost associated with the exoskeleton’s own weight. This principle delineates a critical direction for the development of wearable load-bearing exoskeletons. Fourth, Targeted Optimization of the Bowden Cable Routing Path. This study introduces a significant improvement in the layout of the Bowden cable. Unlike conventional designs where the cable is directly anchored to fixed points, the proposed scheme suspends it from the waist region. Although the traditional direct-anchoring approach shortens the cable path, it inevitably causes friction against the thigh surface during operation, and the force transmission direction deviates from the alignment with the thigh’s motion axis. This misalignment converts part of the assistive force into resistive torque, compromising assistance efficiency. By optimizing the routing path, the current design not only maintains high transmission efficiency but also markedly improves kinematic compatibility with natural human movement. Fifth, Enhancement of Knee Joint Stability through Rigid-Flexible Coupled Binding. To address the issue of anterior knee slippage (commonly referred to as “anterior collapse”) in most exoskeletons under load-bearing conditions, this study employs a rigid-flexible coupled binding mechanism. This design integrates a rigid support frame with flexible strapping bands, working synergistically to effectively suppress abnormal displacement of the knee joint during weight-bearing. Simultaneously, the distributed pressure application reduces localized compression, thereby enhancing overall stability while optimizing wearer comfort.

## Figures and Tables

**Table 1:** Physiological Characteristics of Subjects.

Subjects	Age (Year)	Gender	Height (cm)	Weight (kg)
Subject 1	30	Male	186	95
Subject 2	26	Male	176	72
Subject 3	25	Male	176	67
Subject 4	26	Male	179	65
Subject 5	25	Male	173	65
Subject 6	26	Male	173	67
Subject 7	25	Male	170	50
Subject 8	25	Male	172	85

**Table 2:** Evaluation Indicators of Four Prediction Schemes.

Methods	10		15	
	QS	DW	QS	DW
Subject 1	91.29%	50.28%	74.88%	39.63%
Subject 2	95.13%	61.17%	63.62%	46.88%
Subject 3	87.25%	58.31%	70.10%	45.68%
Subject 4	81.70%	77.24%	88.00%	47.06%
Subject 5	88.92%	57.71%	65.48%	39.37%
Subject 6	90.97%	66.62%	63.54%	50.57%
Subject 7	94.33%	67.53%	59.98%	32.00%
Subject 8	94.28%	57.72%	71.99%	48.36%
Mean	90.48%	62.07%	69.70%	43.69%
±SEM	1.59%	2.90%	3.15%	2.18%

All results are presented as mean ± standard deviation.

**Table 3:** Net Metabolic Cost Under Different Conditions.

Subjects	NE	Assist OFF	Assist ON
Subject 1	4.827	4.049	3.749
Subject 2	5.962	5.864	5.268
Subject 3	5.676	5.139	4.293
Subject 4	3.984	3.789	3.233
Subject 5	3.559	3.358	3.029
Subject 6	6.672	5.466	4.776
Subject 7	4.651	3.941	2.758
Subject 8	6.087	6.329	5.373
Mean	5.177	4.742	4.060
±SEM	0.387	0.387	0.361

All results are presented as mean ± standard deviation. All values are expressed in W/kg.

**Table 4:** Comparison of Exoskeletons with Load Transfer Capabilities.

Researcher	Assisted Joint	Load Transfer Ability (%)	Metabolic Cost (%)	Assistance Mode	Total Weight (kg)
Zhou [5]	lower limbs	68.03% & 24.60%	/	Passive	4.5
Zhan [7]	lower limbs	87.80% & 42.41%	−14.33%	Passive	6
Xiang [10]	lower limbs	/	−5.42% & −20.85%	Active	10.8
Zhu [11]	Hip-Knee	/	−10.95%	Active	6.5
Lee [21]	Hip-Ankle	/	−14.88% & −22.03%	Active	9.3
Molinaro [22]	Hip-Knee	/	−5.30% & −19.70%	Active-Passive	7
Cao [23]	Hip	88.00% & 67.00%	−12.8%	Active-Passive	5.6
Yu [24]	Hip	/	−13.1%	Active-Passive	/
This work	Hip	90.48% & 62.07%	−21.61%	Active-Passive	5

## Data Availability

Dataset available on request from the authors.

## Abbreviations

The following abbreviations are used in this manuscript:

Assist OFF	exoskeleton assistance modes OFF
Assist ON	exoskeleton assistance ON
NE	exoskeleton-free condition
RLLEs	Rigid lower limb exoskeletons
NAPLE	non-anthropomorphic passive lower limb exoskeleton
SEAS	series elastic actuators
RSCLLE	Rigid-Flexible Coupled Lower Limb Exoskeleton
